# In Vitro Influence of Specific Bacteroidales Strains on Gut and Liver Health Related to Metabolic Dysfunction-Associated Fatty Liver Disease

**DOI:** 10.1007/s12602-024-10219-1

**Published:** 2024-02-06

**Authors:** Diego Garcia-Morena, Maria Victoria Fernandez-Cantos, Silvia Lopez Escalera, Johnson Lok, Valeria Iannone, Pierluca Cancellieri, Willem Maathuis, Gianni Panagiotou, Carmen Aranzamendi, Sahar El Aidy, Marjukka Kolehmainen, Hani El-Nezami, Anja Wellejus, Oscar P. Kuipers

**Affiliations:** 1 Department of Molecular Genetics, Groningen Biomolecular Sciences and Biotechnology Institute, University of Groningen, Nijenborgh 7, 9747 AG Groningen, The Netherlands; 2 Chr. Hansen A/S, Bøge Allé 10-12, 2970 Hørsholm, Denmark; 3 Friedrich-Schiller Universität Jena, Fakultät für Biowissenschaften, 18K, 07743 Bachstraβe, Germany; 4 School of Medicine, Institute of Public Health and Clinical Nutrition, University of Eastern Finland, 70200 Kuopio, Finland; 5 Department of Microbiome Dynamics, Leibniz Institute for Natural Product Research and Infection Biology (Leibniz-HKI), 07745 Jena, Germany; 6 Department of Medicine and State Key Laboratory of Pharmaceutical Biotechnology, University of Hong Kong, Hong Kong, China; 7 Faculty of Biological Sciences, Friedrich Schiller University, 07745 Jena, Germany; 8 Groningen Biomolecular Sciences and Biotechnology Institute, Host-Microbe Metabolic Interactions, University of Groningen, Nijenborgh 7, 9747 AG Groningen, the Netherlands; 9 Molecular and Cell Biology Division, School of Biological Sciences, University of Hong Kong, Pok Fu Lam, Hong Kong SAR

**Keywords:** MAFLD, Microbiota, Bacteroidales, Antimicrobials, Fatty liver, Probiotics

## Abstract

Metabolic dysfunction-associated fatty liver disease (MAFLD) has become a major health risk and a serious worldwide issue. MAFLD typically arises from aberrant lipid metabolism, insulin resistance, oxidative stress, and inflammation. However, subjacent causes are multifactorial. The gut has been proposed as a major factor in health and disease, and over the last decade, bacterial strains with potentially beneficial effects on the host have been identified. In vitro cell models have been commonly used as an early step before in vivo drug assessment and can confer complementary advantages in gut and liver health research. In this study, several selected strains of the order Bacteroidales were used in a three-cell line in vitro analysis (HT-29, Caco-2, and HepG2 cell lines) to investigate their potential as new-generation probiotics and microbiota therapeutics. Antimicrobial activity, a potentially useful trait, was studied, and the results showed that Bacteroidales can be a source of either wide- or narrow-spectrum antimicrobials targeting other closely related strains. Moreover, *Bacteroides* sp. 4_1_36 induced a significant decrease in gut permeability, as evidenced by the high TEER values in the Caco-2 monolayer assay, as well as a reduction in free fatty acid accumulation and improved fatty acid clearance in a steatosis HepG2 model. These results suggest that Bacteroidales may spearhead the next generation of probiotics to prevent or diminish MAFLD.

**Supplementary Information:**

The online version contains supplementary material available at 10.1007/s12602-024-10219-1.

## Introduction

The gastrointestinal (GI) tract is a complex and dynamic ecosystem. It hosts a diverse population of microorganisms termed the gut microbiota. The exact composition of the gut microbiota varies according to the location in the GI tract, age, diet, chemical exposure, the developing immune system, and, potentially, the founder effects of initial colonizers [1, 2]. The symbiosis between gut microbiota and the host has several effects on GI tract physiology: (1) influencing the mucosal innate and adaptive immune system, (2) providing metabolic functions by assisting the digestion and fermentation of food and producing vitamins and short-chain fatty acids, and (3) providing a defense against pathogens through competition of resources and production of antimicrobial compounds production [3]. Quantitative and qualitative alterations in the gut microbiota can impair this symbiotic relationship, leading, in some cases, to increased GI and vascular permeability. This increased permeability can lead to an increase in the translocation of whole bacteria and bacterial products into the circulatory system, reaching both proximal and distal tissues and contributing to remote tissue injury in metabolic syndrome [3–6]. Liver exposure to these bacteria and bacterial products has been associated with the development of metabolic dysfunction-associated fatty liver disease (MAFLD) [7–9].

MAFLD [10], previously referred to as non-alcoholic fatty liver disease (NAFLD), is an umbrella term that encompasses a broad spectrum of histological conditions. It ranges from steatosis to hepatocellular carcinoma, making it the most common liver disease worldwide and the leading cause of chronic liver disease and hepatocellular carcinoma [11–13]. Moreover, recent reports highlight that the prevalence of MAFLD has risen over the past years and is now estimated to affect over 30% of the population [14, 15], especially in people with type 2 diabetes mellitus (T2DM) and obesity [16–18]. MAFLD has crucial implications for individual and societal health, quality of life, and economic burden. However, its pathophysiology is complex and multifactorial, with multiple risk factors contributing to disease progression, which hampers quick and reliable diagnosis. Several risk factors contributing to MAFLD have been recently addressed [6, 12, 19, 20], and the gut microbiota has been proposed as a crucial player. Concerning associated gut microbiota alterations in MAFLD, it has been observed that the levels of several taxa of the bacterial gut microbiota (BGM) either increase or decrease when comparing patients at different stages of the disease [21]. Consequently, microbiome-related interventions are currently being investigated as a strategy to ensure long-term MAFLD prevention and treatment implementation [9, 22–25].

Antibiotics have been considered a treatment for gut microbiota-related diseases because of their ability to eliminate harmful bacteria in the GI tract. However, it has been shown that the broad-spectrum activity of antibiotics can cause alterations in the microbiota community composition, which subsequently affect microbiota-host and species-species interactions [26–29]. In contrast, narrow-spectrum antimicrobial compounds could be suitable tools to shape the gut microbiota by targeting specific genera without affecting the overall microbiota [30–32]. In addition to antibiotics, bacteriocins could also potentially achieve this goal [33]. Bacteriocins are small, heat-stable, and ribosomally synthesized antimicrobial peptides produced by bacteria competing against other bacteria and for which the producer is immune. These compounds exhibit high diversity in size, structure, mechanism of action, inhibitory spectrum, immunity mechanisms, and target cell receptors [34]. Open-source software such as BAGEL4 [35], antiSMASH [36], and PRISM [37] have been developed for the detection of potential gene clusters associated with the biosynthesis of these antimicrobial peptides.

Bioinformatic analysis revealed an association between MAFLD and several different gut species from the Bacteroidales order [38]. Targeting specific bacterial strains could be a relevant approach to protect against the onset or progression of MAFLD. Therefore, in this study, we focused on using Bacteroidales strains whose genomes encode promising antimicrobial compounds as potential new-generation probiotics [39], as closely related species usually produce narrow-spectrum antimicrobials [40]. Furthermore, to study the bacterial-host interactions in the gut and liver, three different cell line models were applied, including HT-29 cells to assess intestinal inflammatory modulation, Caco-2 cell monolayers to study gut barrier integrity, and HepG2 cells to investigate putative effects on hepatic free fatty acid accumulation.

In this study*, Bacteroides* sp. 4_1_36 (B6) was shown to have antimicrobial activity against *Bacteroides stercoris* DSM 19555, which had previously been correlated with MAFLD [38]. Moreover, strain B6 showed a positive effect on fatty acid clearance in hepatic HepG2 cells as well as potential protective effects on the gut epithelium. B6 significantly prevented tight junction destruction in the presence of two cytokine mixtures, in addition to preventing fatty acid accumulation and promoting fatty acid clearance in the hepatic HepG2 cell line.

## Material and Methods

### Strain Collection

Four bacterial species associated with the MAFLD state referred to as MALFD-associated species (Supplementary Table S1) were obtained from the DMSZ German Collection of Microorganisms and Cultures GmbH (DSMZ ACC 169, Leibniz Institute DSMZ Deutsche Sammlung von Mikroorganismen und Zellkulturen GmbH, Braunschweig, Germany). In addition, the collection of GI tract-associated bacterial strains was completed using strains from the BEI Resources Repository. Detailed information on the bacterial sources of isolation and donor status is presented in Supplementary Table S2. Genome sequences of these strains are available from BEI Resources (https://www.beiresources.org/) and the DSMZ Institute (https://www.dsmz.de/).

### Bacterial Growth Conditions

Bacterial strains were grown at 37 °C in Gifu Anaerobic Medium—GAM) (HiFi Media, EWC Diagnostics) supplemented with 5 µg/mL hemin (Merck Life Science N.V) 0.1M NaOH and 2.5 µg/mL vitamin K1 (Merck Life Science N.V) in 100% EtOH, which serves as a good generalist medium for gut anaerobic bacteria [41, 42]. The bacterial strains were cultured under anaerobic conditions in a Coy Anaerobic chamber (1.5–2.5% H_2_, 5% CO_2_, and 90% N_2_), where O_2_ levels were never above 300 ppm. Agar (Boom B.V.) was added at a concentration of 1.5% when preparing agar plates, and at a concentration of 0.7–0.75% when preparing soft agar. Supplementation with hemin and vitamin K1 was performed after autoclaving the GAM broth, GAM agar, and/or soft GAM agar.

The probiotic strain *Lactocaseibacillus rhamnosus* LGG® was inoculated from frozen stock in De Man, Rogosa, and Sharpe broth pH 6.5 (MRS, Difco™) and cultured overnight at 37 °C. Overnight cultures were sub-cultured as described above using 10-fold dilutions and incubated anaerobically at 37 °C overnight.

Whenever needed, overnight bacterial cultures were collected and washed in PBS 1X (FISHER) twice before resuspending in antibiotic-free Dulbecco’s modified Eagle’s medium (DMEM) (GlutaMAX™, Gibco™, or DMEM with 4.5 g/L glucose, 4 mM L-glutamine, and 110 mg/L sodium pyruvate, VWR) (hereafter ABx), at the appropriate optical density (OD). Cell-free supernatants (CFS) were collected from centrifuged overnight cultures, filter-sterilized, and their pH was adjusted as needed.

### Assessment of the Antimicrobial Activity of Selected Anti-inflammatory Bacteroidales Strains by Spot-on-Lawn or Agar Well Diffusion

To test for antimicrobial compound production, antimicrobial tests were performed, as previously described [43, 44]. Briefly, the bacterial strains were grown anaerobically on supplemented GAM agar plates until visible colonies were formed. Single colony-forming units (CFUs) were dotted onto new plates and incubated overnight. The following day, 10 µL of sensitive strains, grown overnight, was used to inoculate 6 mL soft agar (GAM 0.7% agar supplemented with hemin) and spread over plates containing the potential antimicrobial-producing strains. After 16 h of incubation, the antimicrobial activity was visually assessed by the presence of inhibitory halos surrounding the producer colonies.

The production of antimicrobial compounds has also been studied using CFS. Bacterial strains were cultured in supplemented GAM broth and incubated at 37 °C for 24 h under anaerobic conditions. Bacterial growth was assessed by measuring the optical density at 600 nm (OD_600nm_). CFS from stationary-late stationary cultures was prepared by centrifugation of the grown culture at 10,000 g for 5 min, and then the CFS was filtered through a 0.22 μm cellulose acetate membrane (VWR International B.V.). CFS aliquots were used fresh on the day or stored frozen at − 20 °C until analysis. For antimicrobial analysis, metallic rings of 8 mm diameter were laid on top of supplemented GAM agar plates, which were then overlaid with soft GAM agar (0.7% agar) inoculated with 80 μL of the indicator strains. After the overlay was solidified, the rings were removed and 100 μL of the CFS was placed in the spots left by the rings, followed by 16 h incubation under anaerobic conditions at 37 °C in an upright position to avoid spilling the supernatant. Antimicrobial activity was determined based on the presence of halos around the holes containing each supernatant.

### Immunomodulation on HT-29 Cells

Evaluation of immunomodulation in HT-29 cells was adapted from Kechaou et al. [45] and Pápai et al. [46]. Colorectal cancer HT-29 cells, kindly gifted by Prof. Dr. Steven de Jong (UMCG, Groningen), were grown in DMEM GlutaMAX™ (Gibco™) supplemented with 10% (v/v) heat-inactivated fetal bovine serum (FBS) (Gibco™) and 0.1% (v/v) penicillin/streptomycin (Gibco™) at 37 °C an 5% CO_2_-air atmosphere. The cells were seeded on 24-well plates at a concentration of 5 × 10^5^ cells/well and grown until they reached confluence (7 days ~ 1.83 × 10^6^ cells/well), changing the medium every other day. Prior to co-culture (day 6), the medium was changed to DMEM GlutaMAX™ supplemented with 5% heat-inactivated FBS and without antibiotics. The following day, live bacteria were used to stimulate HT-29 cells. Bacteroidales strains were added to HT-29 cells to a final OD_600nm_ of 0.3 (approx. 3 × 10^7^ CFU/mL). The co-culture was incubated for 6 h with and without tumor necrosis factor (TNF)-α stimulation (5 ng/mL) (Peprotech). After the incubation time, supernatants were collected, centrifuged for 10 min at 13,000 g, and stored at − 20 °C until further analysis. The concentration of pro-inflammatory interleukin-8 (IL-8) was evaluated by two enzyme-linked immunosorbent assay (ELISA) (BioLegend) assays, according to the manufacturer’s instructions. Briefly, a 96-well microplate was coated with 100 μL per well of the diluted capture antibody and incubated overnight at 4 °C. After washing, the microplate was blocked by adding 200 μL of Assay Diluent 1X to each well and incubated at room temperature for 1 h with orbital shaking, followed by washing. Then, 100 μL of sample or standards was added to each well and incubated for 2 h at room temperature with orbital shaking, followed by aspiration and washing. The detection antibody (100 μL) was added to each well and incubated for 1 h at room temperature with orbital shaking, followed by aspiration and washing. Avidin-HRP (100 μL) was added and incubated for 30 min at room temperature with orbital shaking before washing. Substrate solution C (100 μL) was added to each well and incubated in the dark for 30 min, followed by 100 μL of stop solution. The optical density of each well was immediately read using a Varioskan instrument (Thermo Fisher Scientific Inc. MA, USA) microplate reader at 450 nm and 570 nm.

### Transepithelial Electrical Resistance (TEER)

The human colon adenocarcinoma Caco-2 cell line (DSMZ ACC 169, Leibniz Institut DSMZ Deutsche Sammlung von Mikroorganismen und Zellkulturen GmbH, Braunschweig, Germany) was cultured in DMEM (Gibco™) supplemented with fetal bovine serum 20% (v/v) FBS (Gibco™), MEM non-essential amino acids 1% (v/v) (Biowest), and Penicillin–Streptomycin-Amphotericin B 1% (v/v) (Biological Industries), hereafter referred to as complete DMEM. Cells were grown in T75cm^2^ culture flasks (Thermo Scientific 174952) in a humidified atmosphere 37 °C with 5% CO_2_. When Caco-2 cells reached 80% confluence, they were treated with TrypLE Express Enzyme (Gibco, 12604) and sub-cultured into a clean T75 flask. The cell passage numbers ranged from 6 to 30 for this assay.

TEER measurements were performed as previously described [47]. Accordingly, 21 days before the experiment, confluent Caco-2 cells were treated with TrypLE Express Enzyme and seeded apically at 1 × 10^5^ cells/well onto 12-well, 12 mm Transwell® with 0.4 μm pore polyester membrane inserts (Corning). The growth medium was renewed every second day using complete DMEM. To allow for the determination of baseline TEER, Transwell inserts were transferred to CellZscope2 (Nanoanalytics, Munster, Germany) the day prior to the TEER experiment, and the medium was fully replaced by adding 800 μL DMEM ABx medium to the apical and 1.5 mL to the basolateral compartment. The CellZscope2 was maintained in a humidified atmosphere at 37 °C with 5% CO_2_, with hourly readings in each well. This would serve as a baseline for the quality control of stable electrical resistance.

On the day of the TEER run, overnight Bacteroidales cultures were collected and washed twice in PBS before resuspending in ABx at OD_600nm_ = 4. Cell-free supernatants (CFS) were collected from centrifuged overnight cultures, filter-sterilized, and pH adjusted as needed.

For the *L. rhamnosus* LGG^@^ strain, cultures in late exponential/early stationary phase were pooled and centrifuged at 4500 rpm for 10 min at 20 °C to collect the bacterial pellet. The pellet was washed twice in PBS before final resuspension in warm complete ABx-free DMEM at OD_600_ = 4.

Next, CellZscope2 was paused, and 100 μL of apical medium was replaced with either bacterial cells or bacterial supernatant. The final OD_600_ value of the bacterial suspensions was 0.5 (approx. 5 × 10^7^ CFU/mL for the Bacteroidales strains and 1.25 × 10^8^ CFU/mL for the *L. rhamnosus* LGG^@^ strain). Bacterial supernatants neutralized to pH 7.0 were added at a final concentration of 12.5%. Each condition was measured in triplicates. The CellZscope2 was transferred back to the incubator, and readings were resumed for a total of 18 h. Changes in TEER during bacterial stimulation were calculated relative to the latest value recorded immediately prior to stimulation (baseline measurements were set to 100%). The area under the curve (AUC) was calculated for each condition.

For the cytokine cocktail experiment, Caco-2 cells were seeded and treated, as previously described. On the day of the experiment, two pro-inflammatory cocktails were added to Abx-free DMEM at 37 °C: (1) TNF-α (10 ng/mL) and interferon (IFN)-γ (10 ng/mL); (2) TNF-α (100 ng/mL) and interferon IFN-γ (10 ng/mL). Both mixtures were mixed in DMEM Abx-free and pre-warmed to 37 °C. Basolateral media was removed, and fresh Abx medium (1.5 mL) containing the pro-inflammatory cocktail was added to each well. Live bacteria or neutralized CFS from B6 and Parme1 were added to the apical side of the wells. Immediately after adding the cytokine cocktails and the bacteria/media, CellZscope2 was transferred back to the incubator and readings were resumed for a total of 18 h. Changes in TEER during bacterial stimulation were calculated relative to the latest value recorded immediately prior to the stimulation (baseline measurement was set to 100%). The area under the curve (AUC) was calculated for each condition.

### Immunostaining

Following TEER measurements, the cultured Caco-2 cells were transferred to 12-well plates containing PBS 1X. Each Transwell was carefully washed once with PBS 1X, and cell membranes were fixed with 4% formaldehyde (VWR) for 6 min at room temperature (RT) and subsequently washed three times in PBS 1X. Each fixed membrane was removed from the Transwell using a scalpel and transferred to a 48 well-plate (Costar 3548) with PBS 1X. Cell permeabilization was achieved by treatment with 0.2% Triton X-100 (Sigma) for 5 min at RT, followed by a single washing step with PBS 1X. The membranes were blocked for 1 h with 2% bovine serum albumin (BSA; Sigma A4503). Primary antibodies were diluted in BSA, as described in Table 1, and the treated membranes were incubated overnight at 4 °C on a platform rocker (Mimetas, organo-Flow) at 12 14° inclinations per minute (5 s intervals). The membranes were washed three times with 0.25% BSA and 0.1% Triton X-100 in PBS 1X (washing buffer) and incubated for 2 h in the dark at RT with secondary antibodies (diluted in washing buffer according to the descriptions in Table 1). Following incubation with the antibodies, the membranes were washed twice with PBS 1X and labeled with nuclei staining 4′,6-diamidino-2-phenylindole (DAPI, Life Technologies D1306, 1:1000 dilution) and incubated for 5 min, protected from light. The membranes were washed thrice with deionized water. Upon completion of the immunofluorescence protocol, membranes were transferred onto 18 mm slides (VWR European 631–1550), mounted with fluorescence mounting medium (Agilent S3023) and coverslips (Karl Hecht GmbH). Staining was visualized using an EVOS™ FL Auto 2D fluorescence microscope (Invitrogen) at 40 × magnification. Bioimaging analysis was performed using Fiji (ImageJ) software (version 2.9.0/1.53t) as described by Shihan et al. [48]. Occludin corresponding channel staining (Alexa Fluor 488) was selected as the area of interest, and the background was subtracted using a rolling ball radius set at 30 pixels. Furthermore, the mean fluorescent intensity (MFI) of the area of interest was measured by subtracting the MFI of the background (calculated as the average of three distinct regions in the background of the area of interest). The MFI was calculated for one sample per condition; therefore, MFIs were not representative of the full experiment.

**Table 1:** Antibodies used in the immunostaining preparation

Antibody/protein	Function	Dilution	Product ID
Mouse anti-human occludin	Tight junction marker primary antibody	1:100	Thermo Fisher 61–7300
Goat anti-mouse, Alexa flour 488	Secondary antibody DAPI 1:1000	1:1000	Life Technologies A-11001

### HepG2, MAFLD Induction, and Treatment

The human hepatocellular carcinoma cell line HepG2 (HB-8065) was purchased from the American Type Culture Collection (ATCC) (Manassas, VA, USA). The cells were cultured in DMEM with 4.5 g/L glucose, 4 mM L-glutamine, 110 mg/L sodium pyruvate (VWR), 10% (v/v) heat-inactivated FBS, and 1% (v/v) Penicillin–Streptomycin-Amphotericin B Solution (Fisher Scientific). The cell cultures were maintained in a T75cm^2^ culture flask at 37 °C in an incubator with a controlled humidified atmosphere containing 5% CO_2_. When the HepG2 cells reached 70% confluence, they were detached from the culture flask using Trypsin–EDTA (0.5 mg/mL trypsin and 0.2 mg/mL EDTA—disodium ethylenediaminetetraacetic acid) and seeded according to the experimental conditions. The cell passage numbers ranged from 10 to 26 for this assay.

The in vitro steatosis model was performed as previously described [49]. Briefly, free fatty acids (FFAs), palmitic acid, and oleic acid were dissolved in 100% ethanol to a final concentration of 100 mM each and filter-sterilized. The final mixture of FFAs used to trigger steatosis was composed of palmitic acid and oleic acid in a 2 to 1 ratio (2 palmitic acid:1 oleic acid). To prepare the steatogenic medium, the FFA mixture was added to incomplete DMEM supplemented with 1% fatty acid-free BSA (FAF-BSA) at 500 µM, filter-sterilized, and used for steatosis induction.

Confluent HepG2 cells were treated with trypsin–EDTA, seeded at 4 × 10^5^ cells/mL in a 24- or 96-well culture plate, and incubated for 24 h at 37 °C with 5% CO_2_. The next day, the medium was replaced with steatogenic medium, prepared as described above. After 24 h, the medium was replaced with either fresh Abx medium (DMEM), fresh complete DMEM containing CFS from either *Bacteroides* sp. 4_1_36 (B6), *Phocaeicola dorei* CL02T12C06 (Bd2), or *Parabacteroides merdae* CL03T12C32 (Parme1) at a final concentration of 6.25% or 50%, or fresh complete DMEM containing Silibinin 10 µM. After 6 and up to 24 h of co-incubation (hereafter: treatment), FFA was quantified via Oil Red O (ORO) staining in 24-well plates, whereas cell viability was evaluated via the MTT assay in 96-well plates.

Additionally, in another experiment, HepG2 cells were treated with trypsin–EDTA and seeded at 4 × 10^5^ cells/mL in a 24- or 96-well culture plate and incubated for 24 h. On the next day, the medium was replaced with fresh Abx medium (DMEM), fresh complete DMEM containing CFS from either *Bacteroides* sp. 4_1_36 (B6), *Phocaeicola dorei* CL02T12C06 (Bd2), or *Parabacteroides merdae* CL03T12C32 (Parme1) at a final concentration of 6.25%, or with fresh complete DMEM containing Silibinin 10 µM. After 6 h of co-incubation (hereafter: pre-treatment), the medium was replaced with steatogenic medium, prepared as described above. After 24 h, FFA was quantified via Oil Red O (ORO) staining in 24-well plates, whereas cell viability was evaluated via the MTT assay in 96-well plates.

### Cell Viability Assay

Cell viability was quantified using MTT (3-(4,5-dimethylthiazol-2-yl)-2,5-diphenyltetrazolium bromide, Fisher Scientific). MTT powder was dissolved in PBS 1X at a concentration of 5 mg/mL, filtered, sterilized, and stored at − 20 °C protected from light until use. On the day of the experiment, 10% MTT solution was prepared using incomplete (FBS- and antibiotic-free) DMEM medium. Next, the 96-well plates were washed with PBS 1X, and 100 µL of the MTT mixture was added to each well, wrapped in foil, and incubated at 37 °C in 5% CO_2_ in a humid atmosphere for 2 h. After incubation, the medium was removed and 150 µL of dimethyl sulfoxide (DMSO) (Sigma-Aldrich (D2650)) was added to each well to dissolve the formazan crystals under constant shaking on an orbital plate shaker for 10 min. Purple formazan was quantitatively measured by absorbance readings using Varioskan (Thermo Fisher Scientific Inc. MA, USA) at 570 and 655 nm as the reference wavelengths. Cell viability was plotted as the percentage of the subtracted OD values of the treated cells compared to the untreated cells as the control (DMEM 0 µM).

### Oil Red O (ORO) Staining

The effect of the treatments on HepG2 lipid content was evaluated by ORO staining (Merck Life Science N.V.). The cells were seeded in 24-well plates, washed twice with PBS, and fixed with 4% formaldehyde (Sigma-Aldrich) for 1 h at RT. Meanwhile, ORO (Sigma-Aldrich (O0625-100G)) working solution was prepared by filtering a mixture of three parts of ORO stock (3 mg/mL ORO in 100% isopropanol) with two parts of distilled water (dH_2_O). The fixed cells were washed with dH_2_O twice and rinsed with 60% isopropanol for 5 min. Next, the cells were stained with ORO working solution for 15 min and then washed with dH_2_O four times. To quantify the ORO content, 100% isopropanol was added to each well before shaking at room temperature on an orbital plate shaker for 5 min, and the isopropanol-extracted sample was then measured at 500 nm using a Varioskan (Thermo Fisher Scientific Inc. MA, USA). ORO was plotted as the ratio (fold of control) between the absorbance value of each of the wells and the average of the OD values of the unsupplemented and untreated cells (DMEM 0 µM).

### Statistical Analysis

Data are expressed as mean ± SDs. Statistical analysis of the results was performed by one-way ANOVA followed by Dunnett’s multiple comparisons test or two-way ANOVA followed by Dunnett’s multiple comparisons test using GraphPad Prism 8.0.2 (GraphPad, San Diego, CA, USA). The results were considered statistically significant at *p* < 0.05.

## Results

### Immunomodulatory Effect and Antimicrobial Profile of a Broad Panel of Bacteroidales Strains

**Fig. 1:**
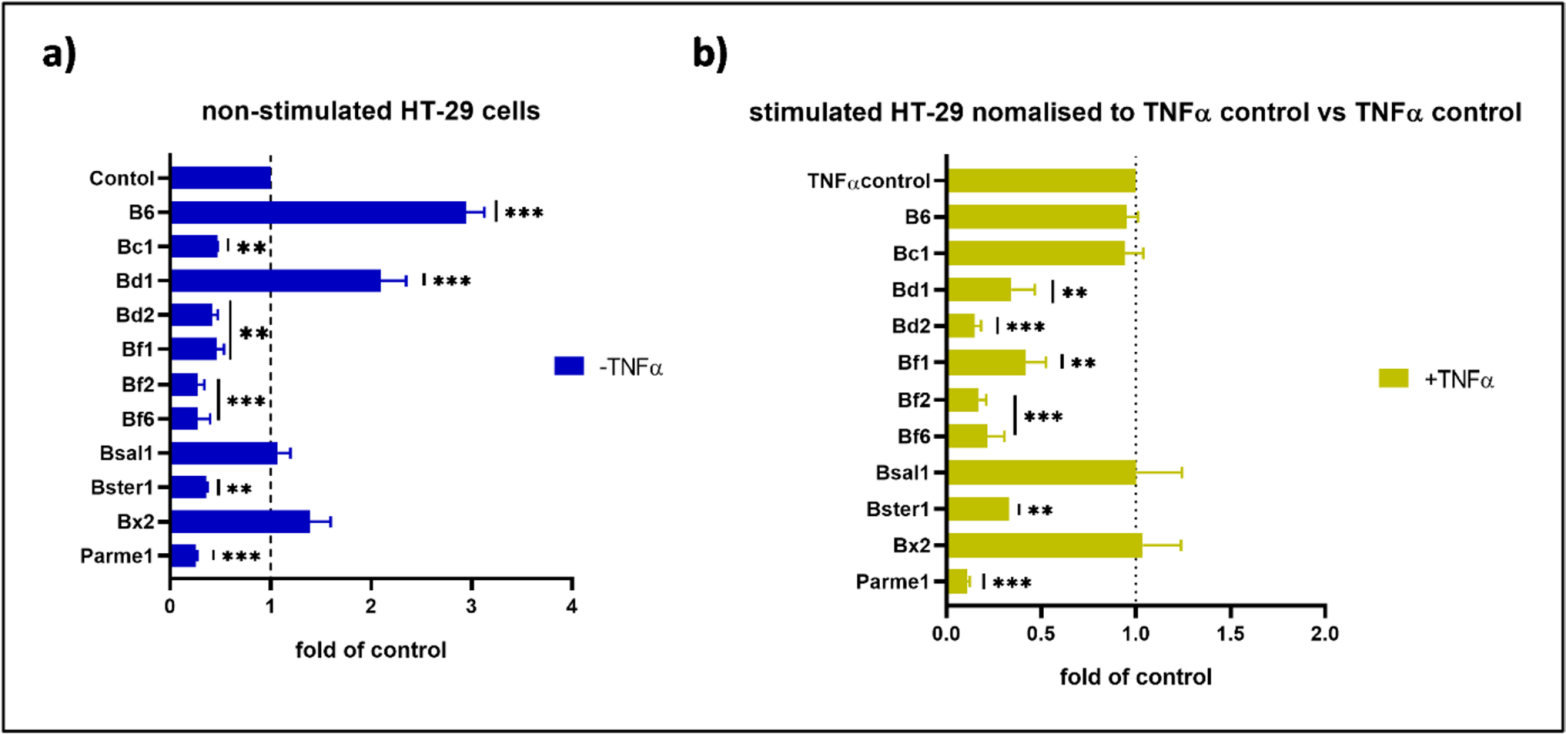
Normalized interleukin-8 (IL-8) levels in non-stimulated **(a)** and in TNFα stimulated **(b)** HT-29 cells when tested with live bacteria from various Bacteroidetes strains. B6, *Bacteroides* sp. 4_1_36; Bc1, *Bacteroides caccae* CL03T12C61; Bd1, *Phocaeicola dorei* CL03T12C01; Bd2, *Phocaeicola dorei* CL02T12C06; Bf1, *Bacteroides fragilis* 3_1_12; Bf2, *Bacteroides fragilis* CL03T12C07; Bf6, *Bacteroides fragilis* NCTC 9343; Bsal1, *Bacteroides salyersiae* DSM18765; Bster1, *Bacteroides stercoris* DSM19555; Bx2*, Bacteroides xylanisolvens* DSM18836; Parme1, *Parabacteroides merdae* CL03T12C32. One-way ANOVA followed by Dunnett’s multiple comparisons test *p* < 0.05: *, *p* < 0.009: **, *p* < 0.001: ***, n = 2 ELISA replicates for all samples

Here, we report the pro- and anti-inflammatory characteristics of HT-29 cells co-incubated with 11 initially selected bacterial strains from the Bacteroidales order of human and mouse origin. Most Bacteroidales strains reduced interleukin-8 (IL-8) production in HT-29 cells in the absence of a pro-inflammatory stimulus (Fig. 1a). In contrast, *Bacteroides* sp. 4_1_36 (B6) and *Phocaeicola dorei* CL03T12C01 (Bd1) significantly increased IL-8 production (*p* < 0.001, Dunnett’s multiple comparison test) in non-stimulated HT-29 cells, suggesting a pro-inflammatory effect on HT-29 cells. TNFα stimulation resulted in a significant, 60-times increase in IL-8 production (baseline control = 49.86 pg/mL, TNFα control = 2919.8 pg/mL). Normalized data showed that several strains were able to significantly reduce IL-8 production in the presence of TNF-α (Fig. 1b). B6 presented a dual role, while seemingly unable to affect IL-8 production in the presence of TNFα, and increased IL-8 secretion in the absence of TNFα stimulation. *P. dorei* CL02T12C06 (Bd2), *B. fragilis* 3_1_12 (Bf1), *B. fragilis* CL03T12C07 (Bf2), *B. fragilis* NCTC9343 (Bf6), *B. stercoris* DSM19555 (Bster1), and *Parabacteroides merdae* CL03T12C32 (Parme1), all able to reduce IL-8 levels in the absence of TNF-α, also showed a putative anti-inflammatory profile when HT-29 cells were challenged with TNF-α. Interestingly, when HT-29 cells were challenged with TNFα, co-incubation with Bacteroidales strains did not further increase the levels of IL-8 after TNFα induction.

**Fig. 2:**
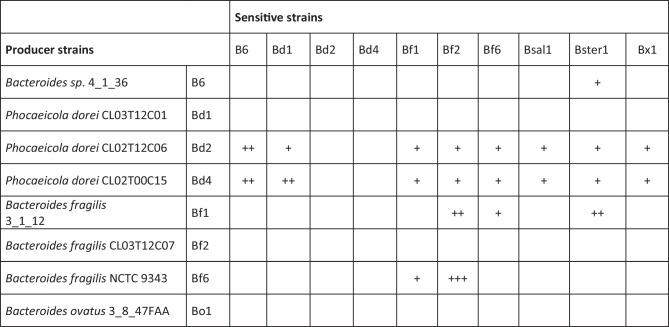
Agar spot overlay assays evaluating antimicrobial inhibition by selected candidates (rows) against putative sensitive Bacteroidales strains (columns). Semiquantitative calculations were made for halo radius, ranging from 100 to 70%: +++, 69 to 30%: ++, and < 29%: +. Empty cells indicate that no halo was found

Although the production of antimicrobial peptides from Gram-positive bacteria has been widely studied, the antimicrobial production capabilities of Gram-negative bacteria are still comparatively unexplored. Interestingly, *P. dorei* CL02T12C06 (Bd2) and *P. dorei* CL02T00C15 (Bd4) displayed a wide range of antimicrobial activities against bacteria, with both pro- and anti-inflammatory properties. In contrast, *B. fragilis* 3_1_12 (Bf1) and *B. fragilis* NCTC 9343 (Bf6) showed narrower antimicrobial activity profiles against members of the same species. *Bacteroides* sp. 4_1_36 (B6) presented a narrow spectrum of antimicrobial activity against *Bacteroides stercoris* DSM 19555 (Bster1), a bacterial strain correlated to MAFLD progression [38]. Remarkably, both *P. dorei* CL02T12C06 (Bd2) and *P. dorei* CL02T00C15 (Bd4) strains showed encouraging antimicrobial potential (Fig. 2) against Bster1 and *Bacteroides xylanisolvens* DSM 10015 (Bx1). Cell-free supernatant (CFS) from selected Bacteroidales strains were also assayed for antimicrobial activity; however, these yielded less pronounced zones of inhibition than those of the whole-cell assays (Supplementary Fig. S1). This could be explained by differences in the concentration of the active antimicrobial compound in CFS compared to that produced by actively growing cells in the agar overlay due to several factors, such as growth phase (unpublished observations) and/or quorum sensing.

Candidate bacterial strains were selected on the basis of their anti-inflammatory potential (Fig. 1) and antimicrobial activity (Fig. 2 and Supplementary Fig. S1). Bd2 and Bf6 were of primary interest, whereas B6 was included because of its specific antimicrobial activity against Bster1, although it showed a pro-inflammatory profile in the absence of TNF-α. A specific inter-species activity could prove more desirable than a wider antimicrobial activity, which could potentially fend off beneficial microbiota strains.

### The Impact of Bacteroidales on Intestinal Permeability is Strain Dependent

**Fig. 3:**
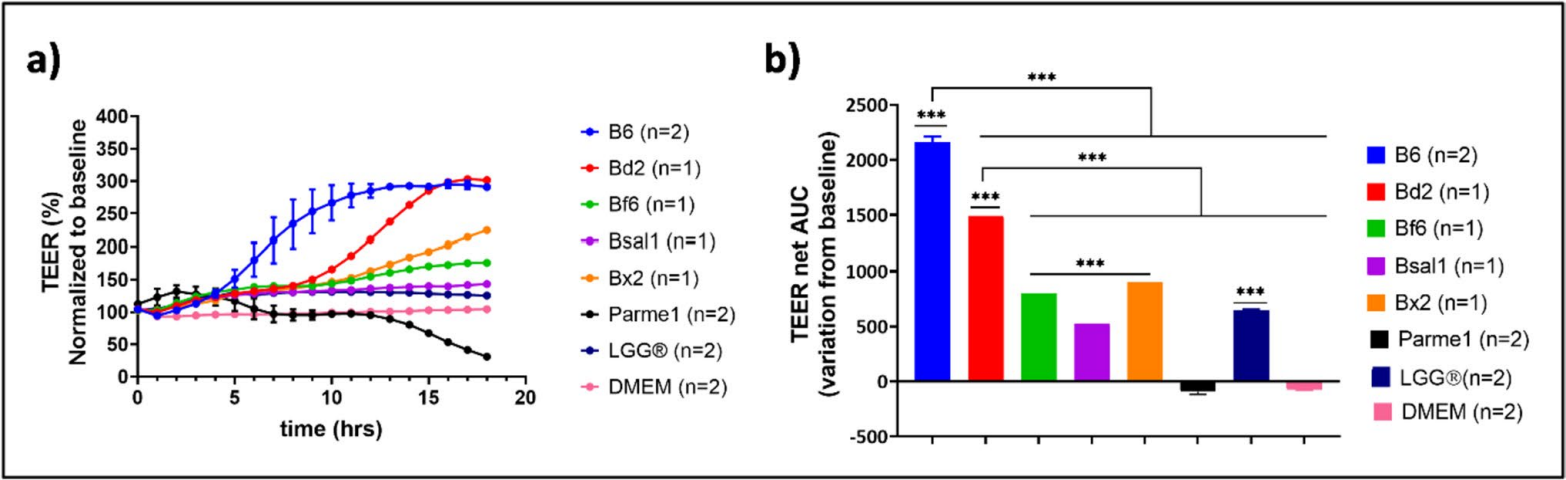
Transepithelial electrical resistance (TEER) readings normalized to baseline measured immediately after adding live bacteria at an OD_600nm_ = 0.3 **(a)**. Area under the curve (AUC) of change from baseline after adding live bacteria **(b)** to the apical side of Caco-2 monolayers. B6, *Bacteroides* sp. 4_1_36; Bd2, *Phocaeicola dorei* CL02T12C06; Bf6, *Bacteroides fragilis* NCTC 9343; Bsal1, *Bacteroides salyersiae* DSM 18765; Bx2, *Bacteroides xylanisolvens* DSM 18836; Parme1, *Parabacteroides merdae* CL03T12C32; LGG, *Lactobacillus rhamnosus*, LGG®; GAM, Gifu Anaerobic Medium. Data shown represent mean + SD (*n* = 3). One-way ANOVA followed by Dunnett’s multiple comparisons test *p* value < 0.001: ***, *n*: biological replicates

Based on the promising outcomes of the antimicrobial assays and the inflammation in HT-29 cells, suggesting the potential beneficial effects of the tested bacteria on human cells, the following experiments focused on assessing the effect of selected Bacteroidales strains on the gut barrier, modeled by Caco-2 cells. Several candidate Bacteroidales strains and their CFS were tested to determine their effects on gut barrier permeability by measuring transepithelial electrical resistance (TEER) values using CellZscope2. The baseline TEER values for Caco-2 cells were stable after 22 h of incubation, reaching an average of approximately 400 Ω⋅cm^2^. Stimulation of Caco-2 cells with viable bacteria significantly changed TEER values (Fig. 3). Among the bacteria assayed, *P. merdae* CL03T12C32 (Parme1) caused a first increase in TEER values, peaking at 3 h, after which the values started to decrease. The TEER reads from Parme1 crossed the baseline after 13 h, suggesting an increased permeability of the Caco-2 monolayer. In contrast, all remaining live bacteria showed significantly increased TEER values (Fig. 3). In particular, *Bacteroides* sp. 4_1_36 (B6) and *P. dorei* CL02T12C06 (Bd2) showed a steady increase in TEER of up to 300% (Fig. 3a). Interestingly, CFS from B6, but not from Bd2, showed a similar effect on the Caco-2 monolayer (Supplementary Fig. S2).

TEER values for the Caco-2 monolayer co-incubated with B6 or Bd2 live bacteria and CFS from B6 or Bd2 were higher than those of the well-characterized probiotic strain LGG® [50] (Fig. 3 and Supplementary Fig. S2). Parme1 displayed negative effects on barrier integrity by decreasing the TEER values after 13 h of incubation (Fig. 3a). Taken together, these results suggest that B6 and Bd2 have a protective effect on the Caco-2 monolayer, potentially by strengthening tight junctions. Although B6 and Bd2 had a positive effect on Caco-2 cells, a significant difference in the performance of both strains was observed, where both live bacteria and CFS from B6 induced significant TEER values. In contrast, the effect of CFS from Bd2 was lower than that of CFS from B6 (Supplementary Fig. S2). The differences in TEER values for both strains could arise from potential differences in the ability to physically adhere/attach to the host, or due to the differences in produced and secreted metabolites. To further study the potential of B6 to impair gut permeability, Caco-2 cells were treated with two pro-inflammatory cytokine mixtures, as well as with CFS of live bacteria from B6. Cytokine treatment significantly reduced TEER, indicating gut barrier permeability (Supplementary Fig. S3). However, co-treatment with B6 (live bacteria or CFS) prevented TEER reduction, suggesting that B6 may act as a protective agent for the gut epithelium. Furthermore, the beneficial effect of B6 in enhancing the integrity of the epithelial barrier was supported by the protein expression of the tight junction occludin observed by immunofluorescence staining (Supplementary Fig. S4). When Caco-2 cells were treated with B6, the mean fluorescent intensity (MFI) of occludin was higher than that of cytokine treatment. Notably, the co-administration of B6 with cytokines resulted in an increase in the MFI of occludin compared with cytokine treatment alone. This suggests a potentially beneficial effect of B6 on inflammatory conditions.

### Establishment of a Free Fatty Acid (FFA)-Induced HepG2 Cell Steatosis Model and the Effect of Bacteroidales Strains on FFA-Induced Lipid Accumulation in Human HepG2 Liver Cells

To establish an FFA-induced HepG2 model, HepG2 liver cells were incubated for 24 h with a 2:1 mixture of oleic acid and palmitic acid. MTT assay was used to determine the effect of the FFA mixture on cell viability, and Oil Red O (ORO) staining was used to quantify FFA accumulation in HepG2 cells. HepG2 cells treated with low FFA concentrations ranging between 0 µM and 500 µM showed no significant loss of cell viability, whereas at 1000 µM, cell viability significantly decreased (Supplementary Fig. S5a). HepG2 cells treated with low FFA concentrations (15.625–250 µM) showed no significant FFA accumulation after ORO quantification, whereas cells supplemented with 500 µM and 1000 µM FFA showed significantly increased FFA accumulation compared to the control (Supplementary Fig. S5b). Cell viability can affect ORO quantification, where lower ORO values could be explained by a loss in the number of cells able to accumulate FFA. To circumvent this, each sample received an “ORO score” (Supplementary Fig. S5c) in accordance with the following formula:

$$a) \quad \quad \mathrm{ORO\;score}=\frac{{\text{ORO}}}{{\text{MTT}}}$$

The increase in the ORO score for a particular FFA concentration was positively correlated with the amount of accumulated FFA (ORO values) and inversely correlated with cell viability (MTT values). Hence, a higher ORO score arises from either higher steatosis, lower cell survival, or a combination of both. Only the ORO score of HepG2 cells supplemented with 1000 µM and 500 µM FFA showed significant differences compared to that of the control samples (DMEM 0 µM). Importantly, the addition of 500 µM FFA significantly increased FFA accumulation without impairing cell viability. Therefore, to mimic FFA-induced steatosis, follow-up experiments were performed with 500 µM FFA.

After establishing an FFA-induced steatosis model in HepG2 cells, the effect of short-term and long-term CFS incubation of candidate strains was tested on the viability and FFA accumulation of HepG2 cells. This analysis aimed to determine the potential of CFS from selected bacteria as a treatment for FFA accumulation. Accordingly, HepG2 cells were incubated for 24 h with DMEM supplemented with different FFA concentrations (500 µM and 0 µM) and then treated with 50 or 6.25% CFS.

FFA supplementation for 24 h followed by 6 h or up to 24 h CFS treatment did not impair cell viability, except for 24 h treatment with 50% CFS from Parme1 (Supplementary Fig. S6). In accordance with the steatosis model (Supplementary Fig. S5), incubation with ABx for 6 h or up to 24 h after 500 µM FFA treatment resulted in significantly increased ORO values in DMEM 500 µM samples across all conditions (Supplementary Fig. S7). Interestingly, treatment with GAM, regardless of the concentration or incubation time, resulted in significantly increased ORO values, whereas CFS treatment, specifically from B6, resulted in similar to control ORO values (Supplementary Fig. S7). This suggests that fresh GAM medium, but not CFS, can impair FFA clearance from HepG2 cells.

**Fig. 4:**
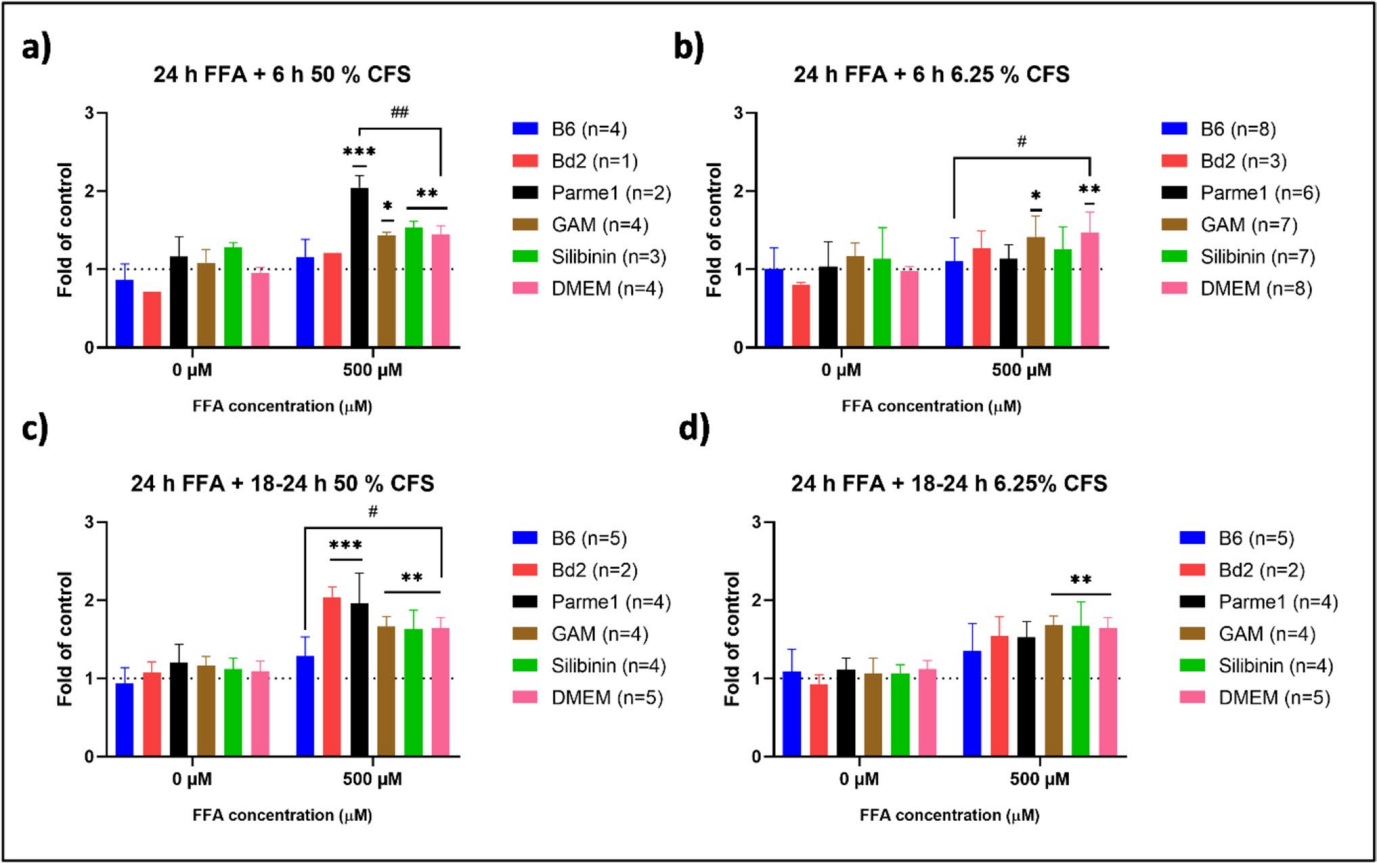
ORO score values, obtained by the ORO to MTT ratio (ORO/MTT) from samples treated for 6 h **(a, b)** or up to 24 h **(c, d)** after FFA supplementation. ORO score values have been normalized to those from untreated, unsupplemented HepG2 cells (DMEM 0 µM). 2-way ANOVA followed by Dunnett’s multiple comparisons test *p* < 0.05: *, *p* < 0.009: **, *p* < 0.001: *** sample groups vs. control group (DMEM 0 µM), *p* < 0.05: ^#^, *p* < 0.009: ^##^, sample group vs. supplemented but untreated cells (DMEM 500 µM). B6, *Bacteroides* sp. 4_1_36; Bd2, *Phocaeicola dorei* CL02T12C06; Parme1, *Parabacteroides merdae* CL03T12C32; GAM, Gifu Anaerobic Medium; DMEM, HepG2 cells without any treatment after FFA supplementation; *n*, biological replicates

However, cell viability can affect ORO values, where its reduction can coincide with the overall lower FFA accumulation quantified by ORO, overlooking the absolute concentration of FFA accumulated per cell (Supplementary Figs. S6 and S7). Advanced cell growth can result in higher ORO values due to the increased number of cells able to accumulate FFA, even if their FFA individual accumulation is lower. Therefore, the population ORO values were corrected for cell viability and are given as an ORO score (ORO/MTT, Fig. 4).

The ORO scores of HepG2 cells supplemented with 500 µM FFA showed significantly increased values compared to those of the control (DMEM 0 µM) (Fig. 4), indicating that these samples accumulated more FFA. Interestingly, across all samples and conditions, only HepG2 cells treated with either 50% or 6.25% B6 CFS resulted in ORO scores similar to those of the DMEM 0 µM control cells (Fig. 4c, d). Furthermore, treatment of HepG2 cells with either 6.25% CFS for 6 h or 50% CFS for 24 h resulted in significantly lower ORO scores compared to those of the supplemented but untreated cells (DMEM 500 µM). This suggests that CFS from B6 can enhance FFA removal by HepG2 cells, despite the detrimental effect of the GAM medium in which B6 was cultured. Moreover, treatment with CFS from B6 outperformed treatment with Silibinin 10 µM, a flavonoid compound used in the treatment of liver diseases [51–53].

While CFS treatment of B6 after FFA supplementation could have beneficial effects on FFA clearance by HepG2 cells, the question remains whether it could have a protective effect, preventing HepG2 cells from accumulating FFA. For this, HepG2 cells were pretreated with 6.25% CFS for 6 h, followed by media change to clean DMEM supplemented with 0 µM and 500 µM FFA for 24 h.

**Fig. 5:**
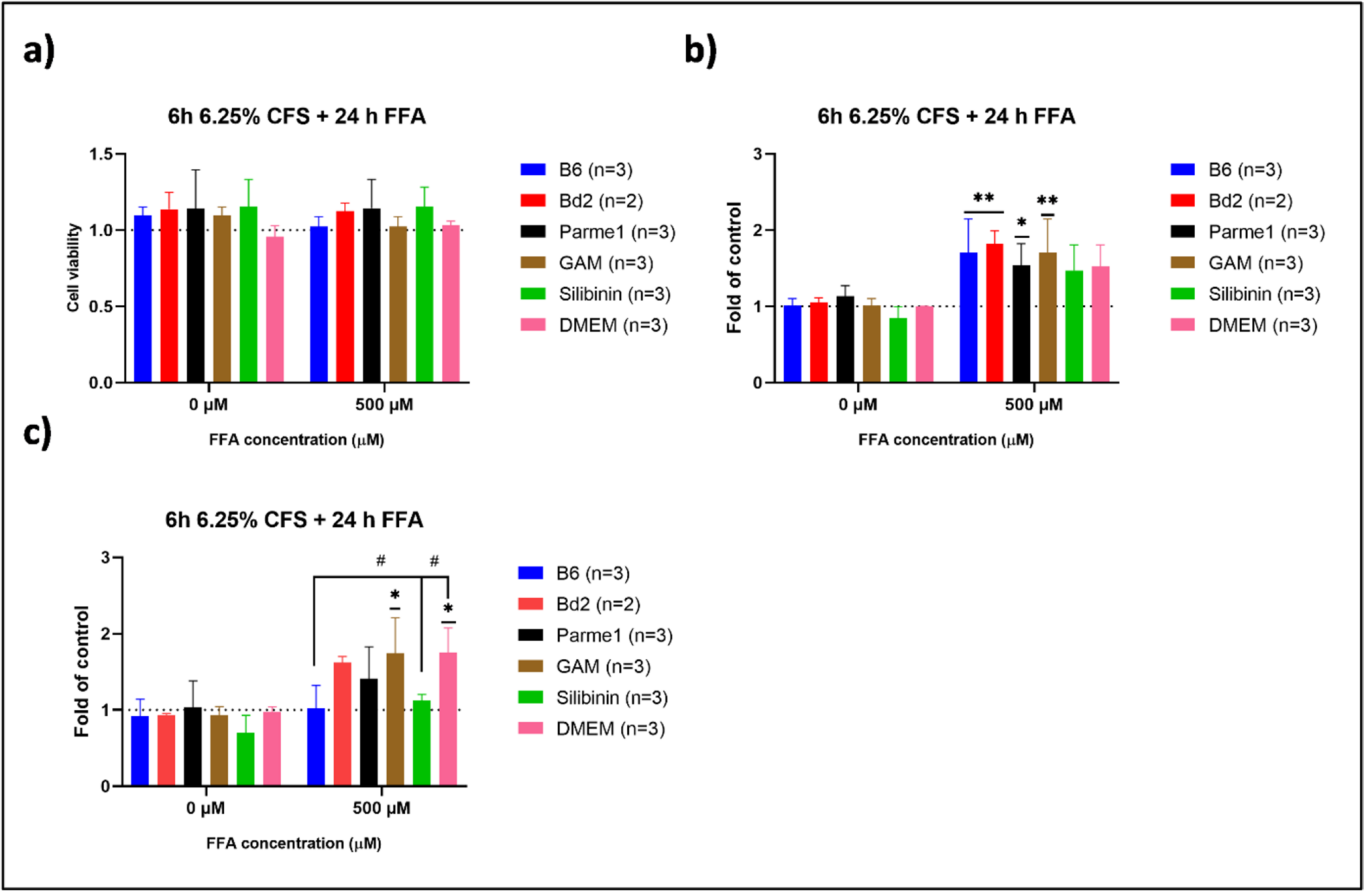
Normalized values of HepG2 cell viability **(a)**, FFA accumulation measured by ORO staining **(b)**, and ORO score **(c)** after 6 h CFS pre-treatment prior to 24 h FFA supplementation. Absorbance (OD_500nm_) has been normalized to untreated, unsupplemented HepG2 cells (DMEM 0 µM). Significance is indicated with horizontal lines as an increase or decrease in ORO values compared to the untreated and unsupplemented cells. Significance between samples is indicated with connecting lines. 2-way ANOVA followed by Dunnett’s multiple comparisons test *p* < 0.05: *, *p* < 0.009: **, sample groups vs. control group (DMEM 0 µM), *p* < 0.05: ^#^, sample group vs. supplemented but untreated cells (DMEM 500 µM). B6, *Bacteroides* sp. 4_1_36; Bd2, *Phocaeicola dorei* CL02T12C06; Parme1, *Parabacteroides merdae* CL03T12C32; GAM, Gifu Anaerobic Medium; DMEM, HepG2 cells without any treatment before FFA supplementation; n, biological replicates

Contrary to CFS treatment, CFS pretreatment resulted in higher general FFA accumulation in all samples supplemented with 500 µM FFA (Fig. 5b). Pretreatment with B6, Bd2, Parme1, and Silibinin did not significantly increase FFA accumulation when HepG2 cells were subsequently supplemented with 500 µM FFA, as evidenced by their lower ORO scores (Fig. 5c). However, only HepG2 cells pretreated with 6.25% CFS from B6 showed significant differences in the ORO score compared to untreated but supplemented cells (DMEM 500 µM). This suggested that CFS from B6 also exerted a significant protective effect, preventing subsequent FFA accumulation in HepG2 cells (Fig. 5c).

In summary, results from the steatosis model showed that treatment or pretreatment with CFS from B6, Bd2, GAM, and Silibinin did not have an impact on FFA accumulation in the absence of FFA supplementation. Remarkably, only treatment with CFS from B6 was able to significantly aid FFA clearance from HepG2 cells supplemented with 500 µM FFA (Fig. 4c, d). This might point to the fact that either the bacterial cells degrade metabolites driving FFA accumulation in HepG2 or that the assayed bacterial cells secrete potentially hepatoprotective metabolite(s). Finally, pre-treatment with CFS from B6 resulted in significantly lower FFA accumulation, suggesting that CFS from B6 could potentially contain prebiotic metabolite(s) (Fig. 5c).

## Discussion

MAFLD is a chronic disease with multiple complex pathological manifestations, where fatty acid accumulation in the liver triggers inflammation and fibrosis, contributing to further liver damage. Obesity, insulin resistance, and other metabolic diseases usually accompany MAFLD [10, 14, 54–56]. Individually tailored behavioral interventions such as dietary changes and physical activity are among the main approaches used to treat patients with MAFLD. However, the expectations of tangible results by patients (e.g., quick weight loss) make lifestyle changes difficult to maintain in the long term [57–60]. The high prevalence of MAFLD and other metabolic disorders and the rapid increase in prevalence over the last decade calls for immediate action in addressing the onset and progression of the disease [10, 15, 61]. The association of the gut microbiota with different diseases, including MAFLD, opens the possibility for microbiome-related interventions as an alternative to classical approaches. However, despite major research efforts into the bacterial gut microbiota, examination of the impact and potential beneficial roles of the Bacteroidetes phylum is, surprisingly, lackluster, even more when considering the high presence of members of this phylum in the gut [62–65]. Notably, antimicrobial activity has been widely studied in Gram-positive bacteria [66]. However, the antimicrobial compounds produced by and/or targeting gut Gram-negative bacterial strains have only recently begun to be elucidated [32, 67–71]. Therefore, the identification of novel antimicrobials from Bacteroidales could alleviate the burden of antibiotic resistance and aid in targeted microbiome modulation, which suggests Bacteroidales strains as putative candidates for targeted microbiota modulation [68, 69]. In this study, *Bacteroides* sp. 4_1_36 (B6) and *Phocaeicola dorei* CL02T12C06 (Bd2) showed inter- and intra-specific antimicrobial activity against members of the same phylum. They also showed potentially beneficial traits in protecting the gut barrier and reducing FFA accumulation in the liver in in vitro cell models.

Gut inflammation has been correlated with increased gut permeability, a trait usually manifested in patients with MAFLD [2, 72–74]. Therefore, a selection from a panel of 11 Bacteroidales strains was made based on their immunomodulatory potential and antimicrobial activity against other members of the Bacteroidales order. Specific antimicrobial activity can be a desirable trait, although the producer strain might have a detrimental effect on gut health, limiting or counteracting the benefits provided. While B6 presented a pro-inflammatory profile, increasing the IL-8 production in HT-29 cells without TNFα stimulation, it showed narrow-spectrum activity, particularly against *B. stercoris* DSM 19555 (Bster1), which had previously been correlated with MAFLD [38]. On the other hand,* P. dorei* CL02T12C06 (Bd2) showed a different inhibition profile, able to inhibit the growth of several genera from the Bacteroidales order, coupled with a strong anti-inflammatory activity. Recent studies have delved into the anti-inflammatory potential of Bacteroidales [75], particularly in *P. dorei* [76], possibly due to their structurally different lipopolysaccharides, modulating the host response [75–78]. Others have hypothesized the role of Bacteroides-derived sphingolipids in gut inflammation [79], as well as on the immunomodulatory effects of outer membrane vesicles produced by *Bacteroides* spp. [80, 81]. The immunomodulatory potential of the selected strains was not a correct indication for a positive or negative effect on Caco-2 permeability. Among the strains with a potential pro-inflammatory profile, both B6 and Bsal1 had a positive impact on the Caco-2 cell monolayer, suggesting a tightening of epithelial integrity. Moreover, B6 showed an increase in TEER values coupled with strengthening of the TJ´s and might have protective effects against the destruction of the TJs, as evidenced by occluding immunostaining. Finally, CFS from B6, Bd2, and Parme1 was tested in a HepG2 steatosis model. Although the pathogenesis of MAFLD is not yet fully understood, it has been established that fatty acid accumulation in the liver and steatosis could be leading drivers of subsequent liver inflammation and fibrosis, resulting in liver damage [21, 23, 54]. In our model, HepG2 cells were treated with 50 or 6.25% CFS from selected Bacteroidales strains after steatosis induction and 6.25% CFS before steatosis induction. Interestingly, fresh GAM medium consistently and significantly increased ORO and ORO scores in the majority of the conditions assayed, while CFS from B6, a strain that had grown in GAM, showed potential as an adjuvant in FFA removal in HepG2 cells. Only HepG2 cells treated with B6 CFS showed significantly lower FFA accumulation in HepG2 cells supplemented with 500 µM FFA, indicating that B6 CFS contributed to FFA clearance from HepG2 cells. Moreover, 6 h pretreatment with 6.25% CFS from B6 prevented FFA accumulation in HepG2 cells supplemented with 500 µM FFA. The significant accumulation of FFA in cells treated with GAM points towards metabolites present in the media could enhance the molecular pathways already altered by the FFA treatment or contribute towards an inflammatory response. The effect of CFS from B6 could be doubled, either by the production and export of molecules able to aid the HepG2 cells in FFA clearance or by the biotransformation of the aforementioned detrimental metabolites present in the GAM medium. Nevertheless, future experiments are needed to establish a molecular reason behind the observed FFA reduction. Future work should dive into the metabolic interaction between the HepG2 cells and the bacteria, as well as in the transcriptome alteration induced by GAM or CFS treatment.

Despite the promising applications of the strains studied, several limitations should be considered. Antimicrobial activity is highly dependent on the growth medium used in the assay. In fact, the range of strains sensitive to a given antimicrobial producer strain can be broadened when performing antimicrobial assays in the presence of non-ionic osmotic pressure (Supplementary Fig. S8). However, they remained unchanged in the presence of ionic osmotic pressure (unpublished observations). Gut microbiota may be subjected to rapid changes in environmental factors, such as osmotic pressure, antibiotics, and pH, which would force the adoption of different strategies to ensure survival. In particular, osmotic pressure has been shown to be a regulated factor associated with elevated antioxidant levels in the gut [82], as well as an antibiotic resistance modulator in the gut [83]. Interestingly, ionic osmotic stress made *Bacillus subtilis* cells insensitive to positively charged antibiotics, most likely due to a decreased affinity to the cell’s membrane [84]. Not only the bacteria but also non-penetrating polymers (such as dietary fibers) can osmotically collapse or dehydrate the mucus network [85], facilitating physical contact between the gut microbiota and the host. In vitro studies, although highly used in research, present limitations such as changes in phenotype, cell function, and gene expression may influence the interpretation of results. Finally, while this study is aimed at identifying potentially relevant Bacteroidales strains, it did not specifically analyze the mechanism of action between the interaction of such strains and cell models, opening the possibility for future work on this matter.

In summary, increasing evidence suggests that the gut microbiota plays a major role in MAFLD as a driver of disease but also as a protective agent. To our knowledge, this is the first study to report that *Bacteroides* sp. 4_1_36 (B6) and *Phocaeicola dorei* CL02T12C06 (Bd2) showed an increase in epithelial integrity in Caco-2 cells, as evidenced by high TEER values, while exerting a putative immunomodulatory effect on HT-29 cells. Additionally, CFS from B6 showed the ability to aid FFA clearance and protection from subsequent FFA accumulation in a HepG2 cell steatosis model. Thus, it has potential as a new probiotic strain for gut and liver health.

## Supplementary Information

Below is the link to the electronic supplementary material.

Supplementary file1 (DOCX 20 KB)

Supplementary file2 (DOCX 1.34 MB)

## Data Availability

The authors confirm that the data supporting the findings of this study are available within the article [and/or] its supplementary materials, and the data that support the findings of this study are openly available in the Open Science framework at https://osf.io/y374u/?view_only=bf012eb2d1a64d4f957ebdc9fec360a5.
