# Supplementary material for: In Vitro Influence of Specific Bacteroidales Strains on Gut and Liver Health Related to Metabolic Dysfunction-Associated Fatty Liver Disease
Source: Probiotics Antimicrob Proteins. 2024 Feb 6;17(3):1498–512. doi: 10.1007/s12602-024-10219-1 (PMC12055940; doi:10.1007/s12602-024-10219-1)
Supplement: Supplementary file 1 — Supplementary file1 (DOCX 20 KB) [file 12602_2024_10219_MOESM1_ESM.docx]

*In-vitro* influence of specific Bacteroidales strains on gut and liver health related to Metabolic dysfunction-associated fatty liver disease

Diego Garcia-Morena^1^, Maria Victoria Fernandez-Cantos^1^, Silvia Lopez Escalera^2,3^, Johnson Lok^4^, Valeria Iannone^4^, Pierluca Cancellieri^1^, Willem Maathuis^1^, Gianni Panagiotou^5,6,7^, Carmen Aranzamendi^8^, Sahar El Aidy^8^, Marjukka Kolehmainen^4^, Hani El-Nezami^9^, Anja Wellejus^2^ and Oscar P. Kuipers^1,^*

^1^ Department of Molecular Genetics, Groningen Biomolecular Sciences and Biotechnology Institute, University of Groningen, Nijenborgh 7, 9747 AG Groningen, The Netherlands

^2^ Chr. Hansen A/S, Bøge Allé 10-12, 2970 Hørsholm, Denmark

^3^ Friedrich-Schiller Universität Jena, Fakultät für Biowissenschaften, Bachstraβe 18K, 07743 Jena, Germany

^4^ School of Medicine, Institute of Public Health and Clinical Nutrition, University of Eastern Finland, 70200 Kuopio, Finland

^5^ Department of Microbiome Dynamics, Leibniz Institute for Natural Product Research and Infection Biology (Leibniz-HKI), 07745, Jena, Germany

^6^ Department of Medicine and State Key Laboratory of Pharmaceutical Biotechnology, University of Hong Kong, Hong Kong, China

^7^ Friedrich Schiller University, Faculty of Biological Sciences, Jena, 07745, Germany

^8^ Host-Microbe Metabolic Interactions, Groningen Biomolecular Sciences and Biotechnology Institute, University of Groningen, Nijenborgh 7, 9747 AG Groningen, the Netherlands

^9^ Molecular and Cell Biology Division, School of Biological Sciences, University of Hong Kong, Hong Kong SAR

* Corresponding author: [o.p.kuipers@rug.nl](mailto:o.p.kuipers@rug.nl)

**Supplementary Table S1.** MAFLD-associated bacteria..

| Bacterial strain | DSM deposit | isolation source | Donor status | Type strain |
| --- | --- | --- | --- | --- |
| *Bacteroides xylanisolvens* | DSM 100015 | Wild mouse | Unknown | No |
| *Bacteroides xylanisolvens* | DSM 18836 | Human faeces | Healthy | Yes |
| *Bacteroides salyersiae* | DSM 18765 | Human appendix tissue | Unknown | Yes |
| *Bacteroides stercoris* | DSM 19555 | Human faeces | Unknown | Yes |

**Supplementary Table S2.** Bacterial strains used in the screening for potential antimicrobial activity against targeted bacterial strains.

| Bacterial strain | Code | Source | Donor status | Type strain |
| --- | --- | --- | --- | --- |
| *Bacteroides* sp. 4_1_36 | B6 | Ileum | Crohn’s disease | No |
| *Phocaeicola dorei* CL03T12C01 | Bd1 | Faeces | Healthy | No |
| *Phocaeicola dorei* CL02T12C06 | Bd2 | Faeces | Healthy | No |
| *Phocaeicola dorei* CL02T00C15 | Bd4 | Faeces | Healthy | No |
| *Bacteroides fragilis* 3_1_12 | Bf1 | Colon | Healthy | No |
| *Bacteroides fragilis* CL03T12C07 | Bf2 | Faeces | Healthy | No |
| *Bacteroides fragilis* NCTC 9343 | Bf6 | Appendix | Unknown | Yes |
| *Bacteroides ovatus* 3_8_47FAA | Bo1 | Colon | Crohn’s disease | No |
| *Bacteroides salyersiae* DSM 18765 | Bsal1 | Appendix | Unknown | Yes |
| *Bacteroides stercoris* DSM 19555 | Bster1 | Faeces | Unknown | Yes |
| *Bacteroides xylanisolvens* DSM 18836 | Bx2 | Faeces | Healthy | Yes |
| *Parabacteroides merdae* CL03T12C32 | Parme1 | Faeces | Healthy | No |
