# Supplementary material for: In Vitro Influence of Specific Bacteroidales Strains on Gut and Liver Health Related to Metabolic Dysfunction-Associated Fatty Liver Disease
Source: Probiotics Antimicrob Proteins. 2024 Feb 6;17(3):1498–512. doi: 10.1007/s12602-024-10219-1 (PMC12055940; doi:10.1007/s12602-024-10219-1)
Supplement: Supplementary file 2 — Supplementary file2 (DOCX 1.34 MB) [file 12602_2024_10219_MOESM2_ESM.docx]

*In-vitro* influence of specific Bacteroidales strains on gut and liver health related to Metabolic dysfunction-associated fatty liver disease

Diego Garcia-Morena^1^, Maria Victoria Fernandez-Cantos^1^, Silvia Lopez Escalera^2,3^, Johnson Lok^4^, Valeria Iannone^4^, Pierluca Cancellieri^1^, Willem Maathuis^1^, Gianni Panagiotou^5,6,7^, Carmen Aranzamendi^8^, Sahar El Aidy^8^, Marjukka Kolehmainen^4^, Hani El-Nezami^9^, Anja Wellejus^2^ and Oscar P. Kuipers^1,^*

^1^ Department of Molecular Genetics, Groningen Biomolecular Sciences and Biotechnology Institute, University of Groningen, Nijenborgh 7, 9747 AG Groningen, The Netherlands

^2^ Chr. Hansen A/S, Bøge Allé 10-12, 2970 Hørsholm, Denmark

^3^ Friedrich-Schiller Universität Jena, Fakultät für Biowissenschaften, Bachstraβe 18K, 07743 Jena, Germany

^4^ School of Medicine, Institute of Public Health and Clinical Nutrition, University of Eastern Finland, 70200 Kuopio, Finland

^5^ Department of Microbiome Dynamics, Leibniz Institute for Natural Product Research and Infection Biology (Leibniz-HKI), 07745, Jena, Germany

^6^ Department of Medicine and State Key Laboratory of Pharmaceutical Biotechnology, University of Hong Kong, Hong Kong, China

^7^ Friedrich Schiller University, Faculty of Biological Sciences, Jena, 07745, Germany

^8^ Host-Microbe Metabolic Interactions, Groningen Biomolecular Sciences and Biotechnology Institute, University of Groningen, Nijenborgh 7, 9747 AG Groningen, the Netherlands

^9^ Molecular and Cell Biology Division, School of Biological Sciences, University of Hong Kong, Hong Kong SAR

* Corresponding author: [o.p.kuipers@rug.nl](mailto:o.p.kuipers@rug.nl)

|  |  | **Sensitive strains** | | | | | | | | | |
| --- | --- | --- | --- | --- | --- | --- | --- | --- | --- | --- | --- |
| **Producer strains** | | B6 | Bd1 | Bd2 | Bd4 | Bf1 | Bf2 | Bf6 | Bsal1 | Bster1 | Bx1 |
| *Bacteroides sp.* 4_1_36 | B6 |  |  |  |  |  |  |  |  |  |  |
| *Phocaeicola dorei* CL03T12C01 | Bd1 |  |  |  |  |  |  |  |  |  |  |
| *Phocaeicola dorei* CL02T12C06 | Bd2 |  | + |  |  |  |  |  |  |  |  |
| *Phocaeicola dorei* CL02T00C15 | Bd4 |  |  |  |  |  |  | + |  |  |  |
| *Bacteroides fragilis*  3_1_12 | Bf1 |  |  |  |  |  | +++ | +++ |  | ++ |  |
| *Bacteroides fragilis* CL03T12C07 | Bf2 |  |  |  |  |  |  |  |  |  |  |
| *Bacteroides fragilis* NCTC 9343 | Bf6 |  |  |  |  |  | +++ |  |  |  |  |
| *Bacteroides ovatus* 3_8_47FAA | Bo1 |  |  |  |  |  |  |  |  |  |  |
| **Supplementary Fig. S1** Cell-free supernatant (CFS) assays evaluating antimicrobial inhibition by selected candidates (rows) against putative sensitive Bacteroidales strains (columns)**.** Semiquantitative calculations were made for halo radius, ranging between 100% to 70%: +++, 69% to 30%: ++, and <29%: +. Empty cells indicate that no halo was found. | | | | | | | | | | | |


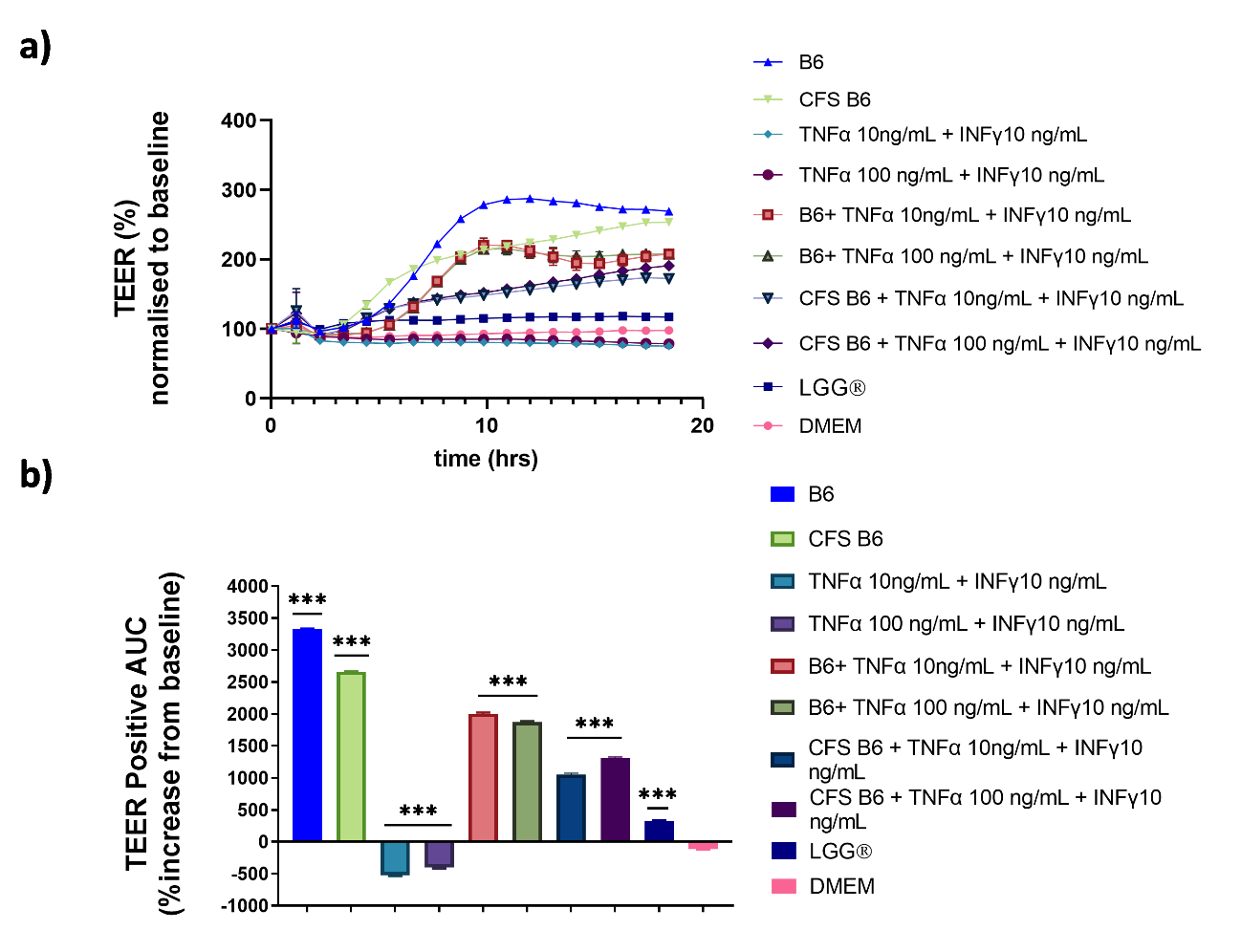


**Supplementary Fig. S2** **a**) 18 h transepithelial electrical resistance (TEER) curve normalized to baseline immediately after adding live bacteria or CFS from B6, two cytokine mixtures and co-treatment with live bacteria or CFS from B6 and either of the two cytokine mixtures. **b**) Area under the curve (AUC) calculated for the treatment of Caco-2 cells with the conditions stated above. One-way ANOVA p-value<0.001: ***. n=1 biological replicate/condition.


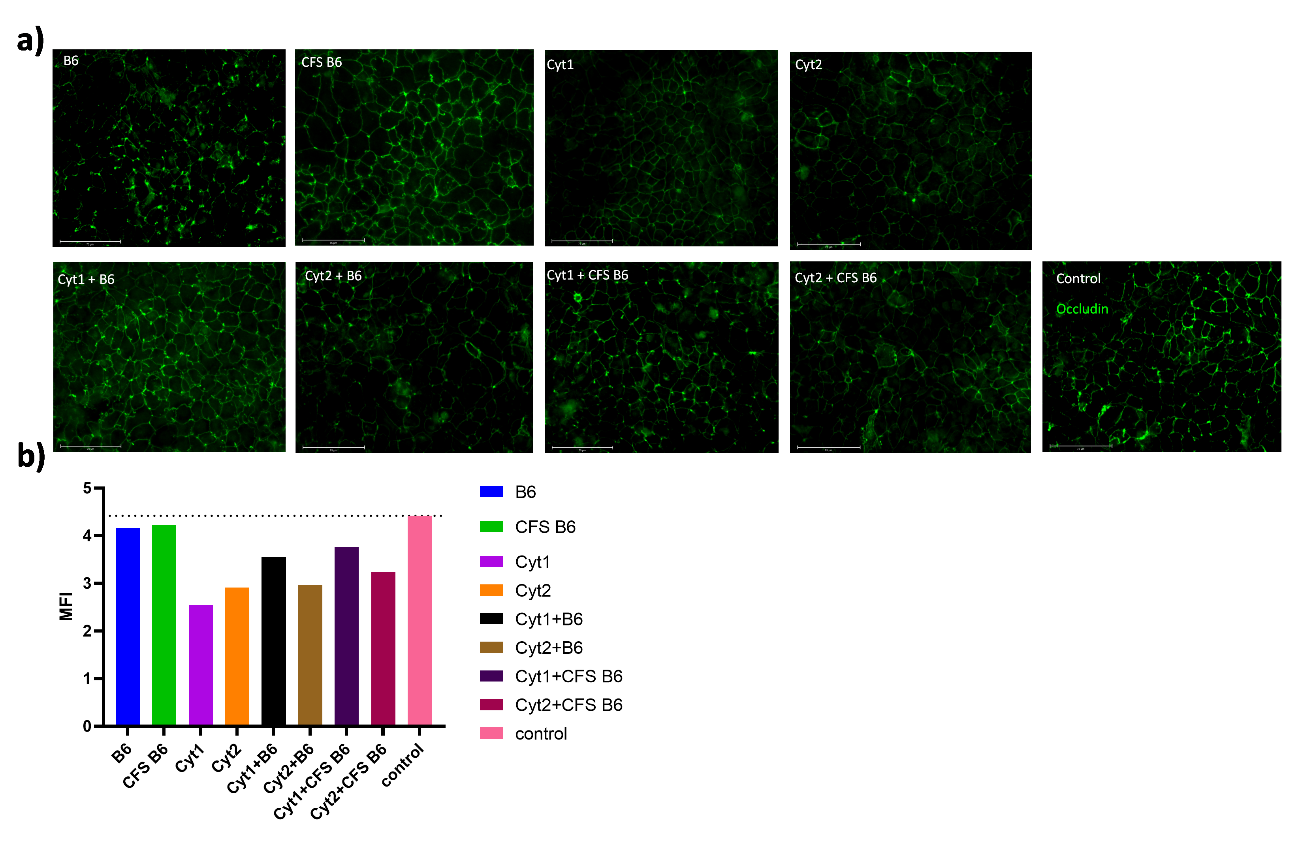


**Supplementary Fig. S3** Occluding expression in Caco-2 cells. **a**) microscope acquisitions of occluding staining in Caco-2 cells at different conditions. B6: *Bacteroides sp.* 4_1_36, Cyt1: TNF-α (10 ng/mL) and interferon (IFN)-γ (10 ng/mL), Cyt2: TNF-α (100 ng/mL) and interferon IFN-γ (10 ng/mL). N=1 biological replicate for all conditions, 40x objective. **b**) bar plots showing the MFI of the representative sample for each condition. N=1 biological replicate for all conditions.


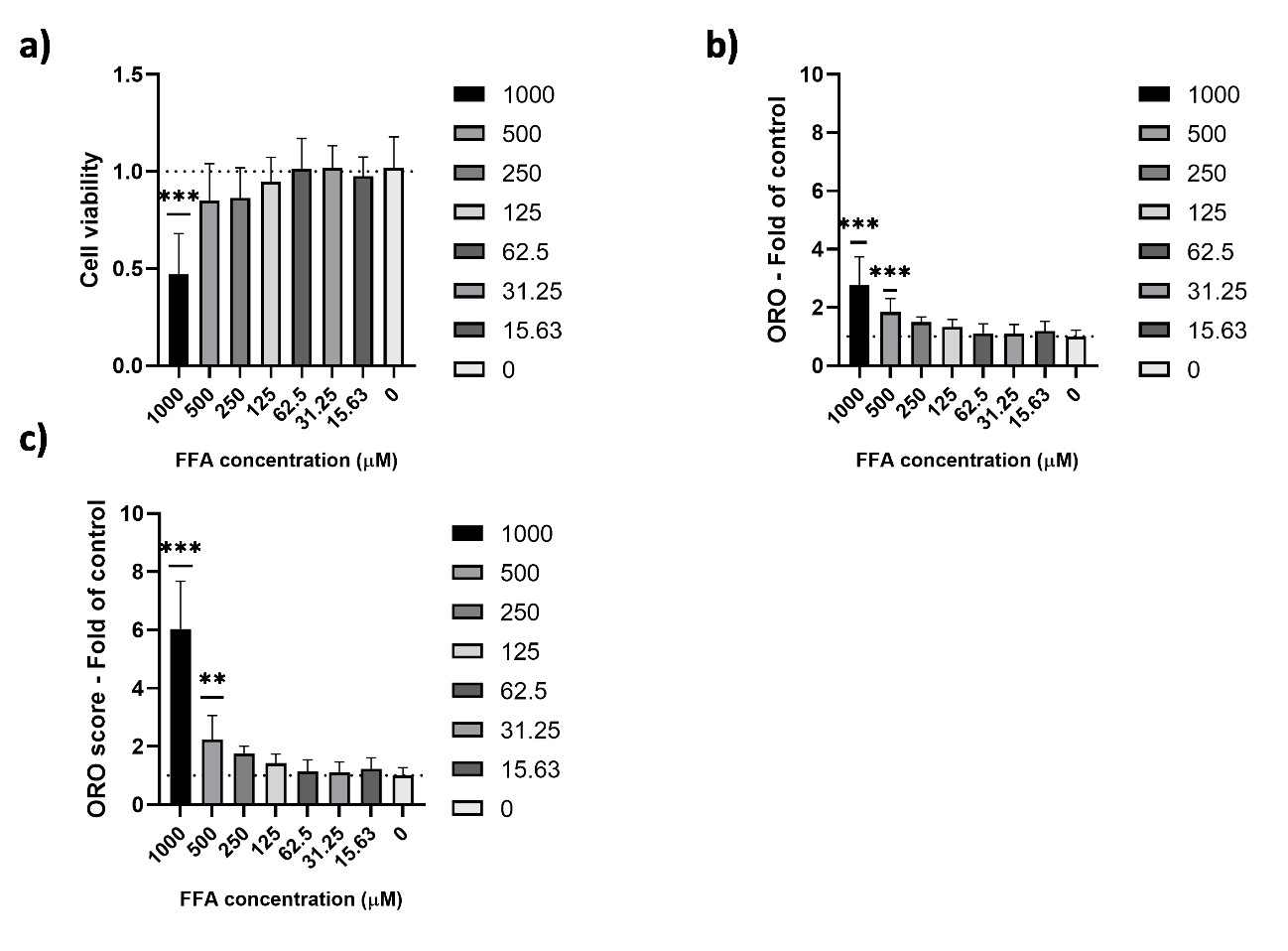


**Supplementary Fig. S4 a**) Normalized viability values after 24 h incubation with different FFA concentrations assayed by MTT. **b**) Normalized Oil Red-O (ORO) quantification at A_500nm_. **c**) ORO score used to correct ORO values against HepG2 cell viability as per the formula ORO score = ORO / MT. One-way ANOVA followed by Turkey’s multiple comparisons test, p value < 0.05: *, p < 0.09: **, p < 0.001: ***. N= 3 biological repliates.


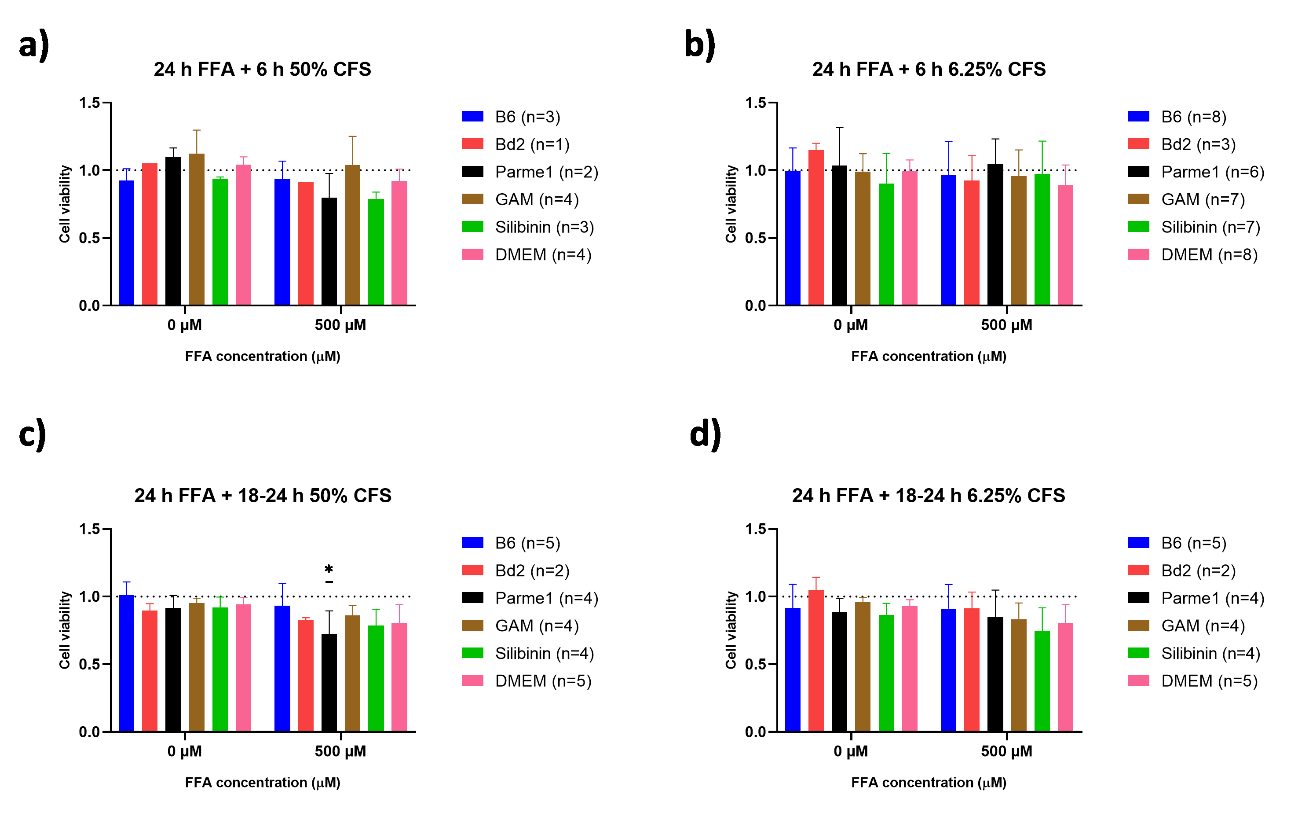


**Supplementary Fig. S5** Normalized values of HepG2 cell viability, measured by MTT, after 6 h (**a**, **b**) or 18 h (**c**, **d**) of cell-free supernatant (CFS) treatment after 24 h free fatty acid (FFA) supplementation. Cell viability was normalised to untreated, unsupplemented HepG2 cells (DMEM 0 µM). 2-way ANOVA followed by Dunnett’s multiple comparisons test p<0.05: *, p<0.009: **, p<0.001:***. B6: *Bacteroides sp.* 4_1_36, Bd2: *Phocaeicola dorei* CL02T12C06, Parme1: *Parabacteroides merdae* CL03T12C32, GAM: Gifu Anaerobic Medium, DMEM: HepG2 cells without any treatment after FFA supplementation.


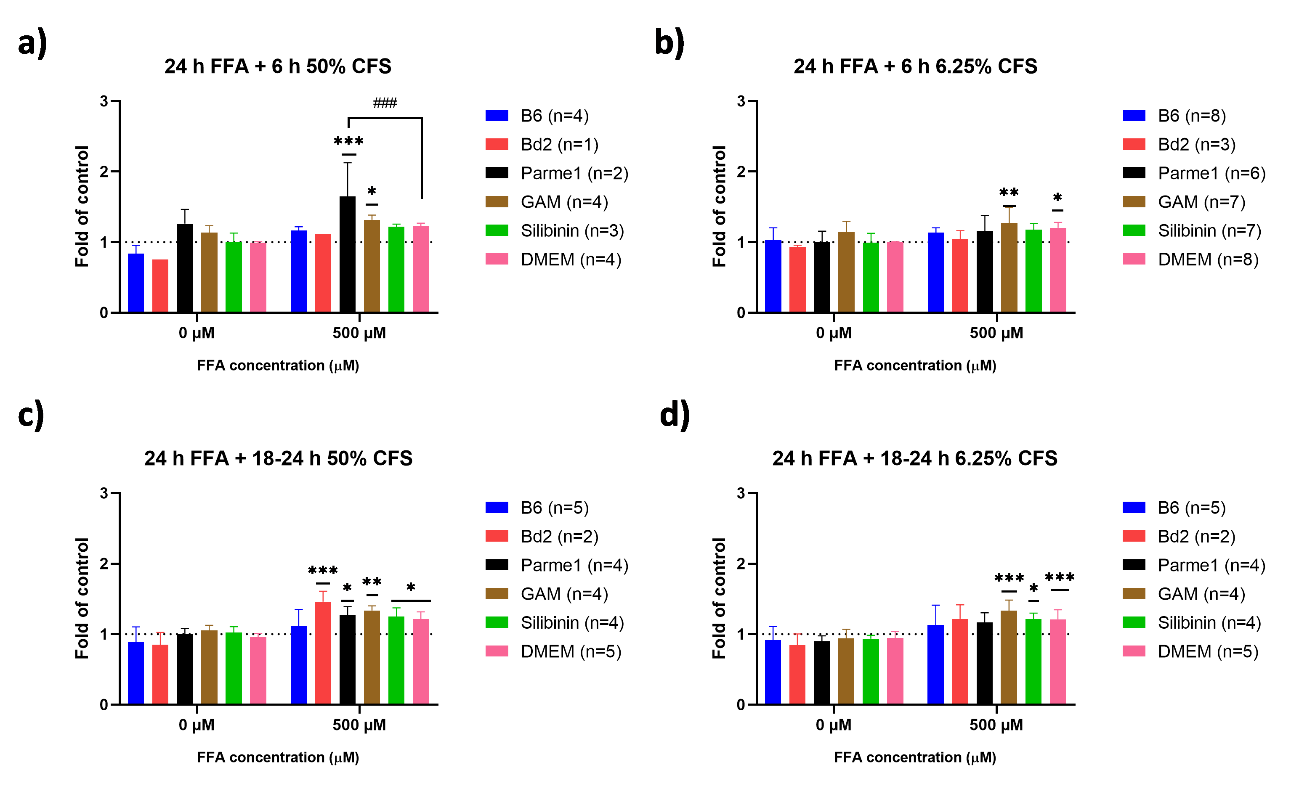


**Supplementary Fig. S6** Normalized values of HepG2 FFA accumulation, measured by ORO staining, after 24 h FFA supplementation followed by 6 h (**a**, **b**) or up to 24 h (**c**, **d**) CFS treatment. Absorbance (OD_500nm_) has been normalized to untreated, unsuplemented HepG2 cells (DMEM 0 µM). 2-way ANOVA followed by Dunnett’s multiple comparisons test p<0.05: *, p<0.009: **, p<0.001: ***, sample groups vs control group (DMEM 0 µM), p<0.001: ^###^, sample group vs supplemented but untreated cells (DMEM 500 µM). B6: *Bacteroides sp.* 4_1_36, Bd2: *Phocaeicola dorei* CL02T12C06, Parme1: *Parabacteroides merdae* CL03T12C32, GAM: Gifu Anaerobic Medium, DMEM: HepG2 cells without any treatment after FFA supplementation, n: biological replicates.

| **Supplementary Figure S7** | **Sensitive strains** | | | | | |
| --- | --- | --- | --- | --- | --- | --- |
| **Producer strains** |  | **Bf1** | **Bf2** | **Bf6** | **Bster1** | **Bx2** |
| Bacteroides fragilis 3_1_12 | Bf1 | + | ++ | + | + |  |
| Bacteroides fragilis CL03T12C07 | Bf2 | + |  |  |  |  |
| Bacteroides fragilis NCTC 9343 | Bf6 | + | +++ | + | + | + |
| Agar spot overlay assays in GAM media containing 0.4M sorbitol. Antimicrobial inhibition by selected candidates (rows) against putative-sensitiv*e Bacteroidetes* strains (columns)**.** Semiquantitative calculations were made for halo radius, ranging between 100% to 70%: +++, 69% to 30%: ++, and <29%: +. Empty cells indicate that no halo was found. | | | | | | |
